# Lack of APLP1 leads to subtle alterations in neuronal morphology but does not affect learning and memory

**DOI:** 10.3389/fnmol.2022.1028836

**Published:** 2022-10-28

**Authors:** Susanne Erdinger, Irmgard Amrein, Michaela Back, Susann Ludewig, Martin Korte, Jakob von Engelhardt, David P. Wolfer, Ulrike C. Müller

**Affiliations:** ^1^Department of Functional Genomics, Institute of Pharmacy and Molecular Biotechnology, Heidelberg University, Heidelberg, Germany; ^2^Institute of Anatomy, University of Zurich and Institute of Human Movement Sciences, ETH Zurich, Zurich, Switzerland; ^3^Institute of Pathophysiology, Focus Program Translational Neuroscience (FTN), University Medical Center of the Johannes Gutenberg University Mainz, Mainz, Germany; ^4^Zoological Institute, Division of Cellular Neurobiology, TU Braunschweig, Braunschweig, Germany; ^5^Helmholtz Centre for Infection Research, AG NIND, Braunschweig, Germany; ^6^Zurich Center for Integrative Human Physiology, University of Zurich, Zurich, Switzerland

**Keywords:** Alzheimer disease, APP, APLP1, amyloid precursor like protein, synaptic plasticity, behavior, learning, memory

## Abstract

The amyloid precursor protein APP plays a crucial role in Alzheimer pathogenesis. Its physiological functions, however, are only beginning to be unraveled. APP belongs to a small gene family, including besides APP the closely related amyloid precursor-like proteins APLP1 and APLP2, that all constitute synaptic adhesion proteins. While APP and APLP2 are ubiquitously expressed, APLP1 is specific for the nervous system. Previous genetic studies, including combined knockouts of several family members, pointed towards a unique role for APLP1, as only APP/APLP1 double knockouts were viable. We now examined brain and neuronal morphology in APLP1 single knockout (KO) animals, that have to date not been studied in detail. Here, we report that APLP1-KO mice show normal spine density in hippocampal CA1 pyramidal cells and subtle alterations in dendritic complexity. Extracellular field recordings revealed normal basal synaptic transmission and no alterations in synaptic plasticity (LTP). Further, behavioral studies revealed in APLP1-KO mice a small deficit in motor function and reduced diurnal locomotor activity, while learning and memory were not affected by the loss of APLP1. In summary, our study indicates that APP family members serve both distinct and overlapping functions that need to be considered for therapeutic treatments of Alzheimer’s disease.

## Introduction

The amyloid precursor protein (APP) is best known for its crucial role in Alzheimer’s disease pathogenesis (De Strooper and Karran, 2016; Haass and Selkoe, 2022), whereas its physiological functions are only beginning to be unraveled (Mockett et al., 2017; Müller et al., 2017). APP belongs to a small gene family of type I transmembrane proteins, which includes in mammals the amyloid precursor like protein 1 and 2 (APLP1 and APLP2) (reviewed in (Müller et al., 2017)). The APP family members show several common features, including complex proteolytic processing by α-, β- and γ-secretase (Scheinfeld et al., 2002; Eggert et al., 2004; Endres et al., 2005; Kuhn et al., 2016; Müller et al., 2017). In addition, APP family proteins share a similar structural organization with conserved E1 and E2 regions located in their large extracellular domains and a highly conserved cytoplasmic tail that links them to a rich network of interacting factors implicated in signaling (Müller et al., 2017). The Aβ peptide, located at the juxtamembrane region is, however, unique for APP.

APP family proteins are highly expressed in neurons, in somata, axons and dendrites and are localized to pre- and postsynaptic sites (Lassek et al., 2013; DeBoer et al., 2014; Del Turco et al., 2016; Schilling et al., 2017), where they function as transsynaptic adhesion molecules that undergo homotypic and heterotypic dimerization (Soba et al., 2005; Kaden et al., 2008; Baumkotter et al., 2014; Stahl et al., 2014; Schilling et al., 2017). Consistent with this, APP family proteins are upregulated both at central and peripheral synapses during postnatal development coinciding with synaptogenesis (Schilling et al., 2017). Whereas APP and APLP2 are ubiquitously expressed in most tissues, APLP1 expression is restricted to neurons (Slunt et al., 1994; Lorent et al., 1995; Thinakaran et al., 1995). At the neuromuscular junction for example, APP and APLP2 are expressed in nerve and muscle, whereas APLP1 is located specifically at innervating axons (Wang et al., 2005, 2009; Caldwell et al., 2013; Klevanski et al., 2014; Schilling et al., 2017).

Gene knockout studies yielded important insights into physiological functions of the APP family for a variety of processes, notably synapse formation, maintenance and plasticity *in vivo*. While mouse mutants lacking only a single family member are fully viable, combined germline APP/APLP2 double knockout (DKO), APLP1/APLP2-DKO and APP/APLP1/APLP2 triple knockout (TKO) mice die shortly after birth (Li et al., 1996; von Koch et al., 1997; Steinbach et al., 1998; Heber et al., 2000; Herms et al., 2004) due to impairments at the neuromuscular junction (Wang et al., 2005, 2009; Weyer et al., 2011; Klevanski et al., 2014; Han et al., 2017) revealing genetic evidence for partially overlapping functions. This is further corroborated by studies of conditional, brain-specific combined mutants (Hick et al., 2015; Richter et al., 2018; Mehr et al., 2020; Steubler et al., 2021). Interestingly, while constitutive germline APLP1/APLP2-DKO mice are lethal, constitutive APLP1/APP-DKO mice proved viable, indicating specific, unique functions for APLP1 (Heber et al., 2000). APLP2-KO mice exhibit normal body weight, grip strength, motor coordination and cognition (von Koch et al., 1997; Weyer et al., 2014). In contrast, APP-KO mice show reduced body weight, reduced grip strength, impaired locomotor activity, impaired passive avoidance and spatial learning, as well as impaired synaptic plasticity (Dawson et al., 1999; Seabrook et al., 1999; Fitzjohn et al., 2000; Ring et al., 2007; Senechal et al., 2008; Zou et al., 2016; Galanis et al., 2021).

Compared to APP, much less is known about the functions of APLP1. Recently, we showed that APLP1 functions as a synaptic cell adhesion molecule with, compared to APP and APLP2, increased transcellular binding and elevated cell-surface levels (Schilling et al., 2017). Interestingly, in AD patients secreted fragments of APLP1 arising due to β-secretase (BACE) processing have recently been identified as a sensitive cerebrospinal fluid biomarker (Dislich et al., 2015; Simoes et al., 2020). In addition, APLP1 has recently been implicated as a possible receptor for α-synuclein fibrils mediating their cell-to-cell transmission (Zhang et al., 2021). As therapeutics targeting APP processing (e.g., BACE and other secretase inhibitors) may also affect APP/APLPs physiological functions, it is thus important to elucidate possible consequences for each APP family member. Here, we studied neuronal morphology in the hippocampus of APLP1-KO mice, examined basal synaptic transmission and synaptic plasticity at the CA3/CA1 pathway, performed a detailed analysis of neuromotor behavior and assessed their performance in various tasks for learning and memory. APLP1-KO mice revealed no deficit in spine density of CA1 neurons and only subtle alterations in dendritic branching. At the behavioral level we report reduced grip strength and impaired locomotor activity, whereas no significant deficits were found in cognitive tasks, consistent with normal synaptic plasticity.

## Results

### Hippocampal pyramidal cells of APLP1-KO mice exhibit normal spine density and only subtle deficits in dendritic branching

**Figure 1:**
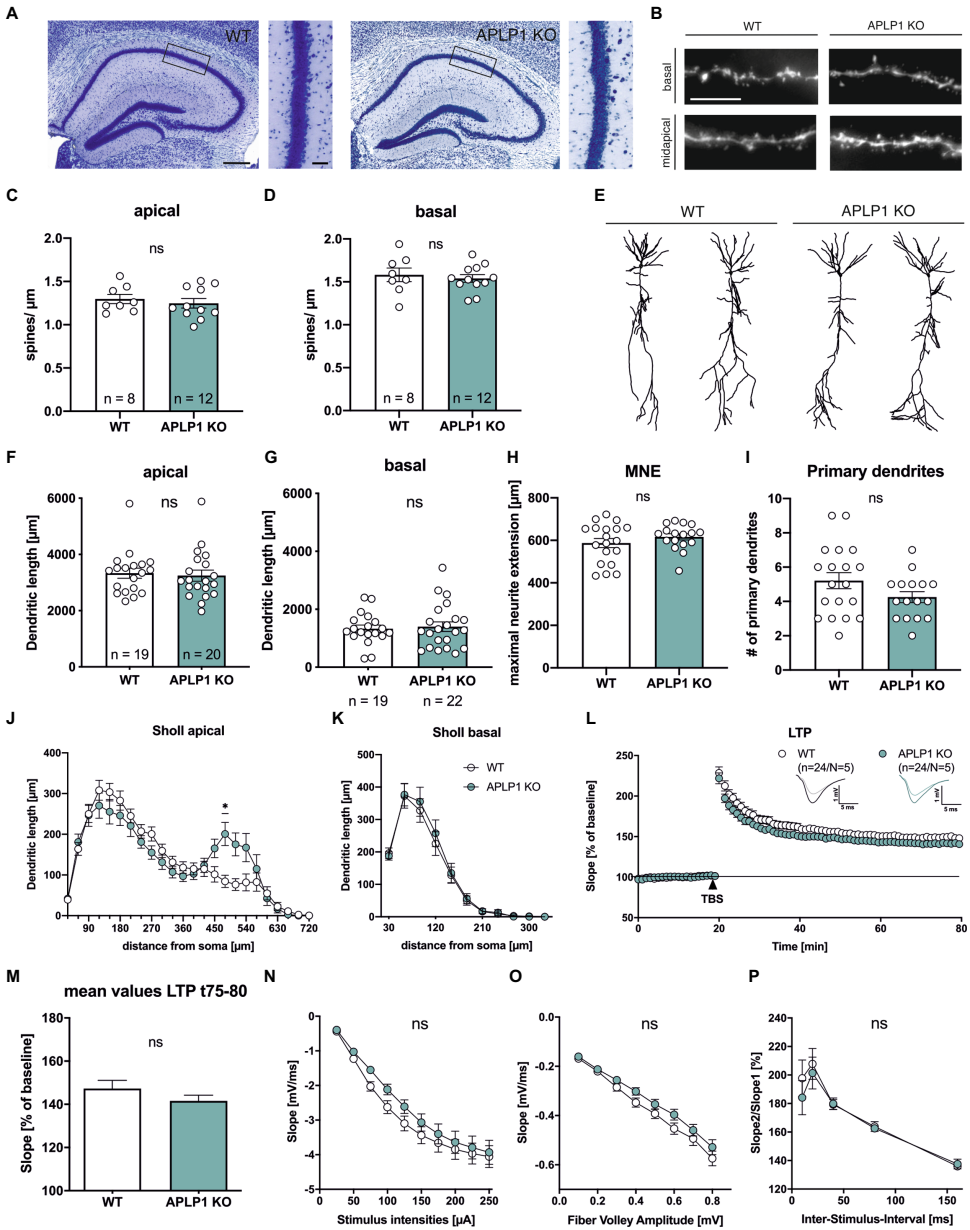
APLP1-KOs show no gross morphological defects and only subtle deficits in neuronal morphology. **(A)**: Giemsa-stained, glycolmethacrylate-embedded coronal brain sections (bregma −1.9) displaying the hippocampus and adjacent callosal fiber tracts of a wildtype WT, left and a APLP1-KO (right; age: 5–6 months). Scale bar: 200 μm, close-up: 50 μm. **(B)**: Representative images of basal and midapical dendritic segments. Brightness and contrast were adjusted to ensure a uniform appearance. Scale bar: 5 μm. **(C, D)**: Spine density of CA1 pyramidal cells is not significantly altered in midapical dendritic segments (C; unpaired Student’s *t*-test, ^ns^p = 0.5306), as well as in basal dendritic segments (D; unpaired Student’s t-test, ^ns^p = 0.6281). **(E)**: Representative 3D reconstructions of CA1 pyramidal neurons from both genotypes. **(F)**: Compared to wildtype controls, CA1 neurons of APLP1-KO animals show no significant reduction in total dendritic length in apical dendrites (Mann–Whitney test, ^ns^p = 0.6667). **(G)**: Compared to wildtype controls, CA1 neurons of APLP1-KO animals show no significant reduction in total dendritic length in basal dendrites (unpaired Student’s t-test, ^ns^p = 0.7506). **(H)**: APLP1-KO neurons show no altered maximal neurite extension (MNE) of apical dendrites compared to wildtype controls (unpaired Student’s *t*-test, ^ns^p = 0.283). **(I)**: The number of primary basal dendrites is significantly unchanged in APLP1-KO animals compared to WT in CA1 (unpaired Student’s *t*-test, ^ns^p = 0.1137). **(J)**: Sholl analysis reveals no genotype effect on apical dendritic segments of CA1 pyramidal neurons (WT: *n* = 19, *N* = 3; APLP1-KO: n = 20/*N* = 4). **(K)**: Sholl analysis reveals no genotype effect on basal dendritic segments of CA1 pyramidal neurons (WT: *n* = 19, *N* = 3; APLP1-KO: *n* = 22/*N* = 4). **(L)**: After 20 min baseline recording, LTP was induced by application of Theta burst stimulation (TBS, arrowhead). Acute slices of APLP1-KO mice displayed an LTP curve that is statistical indistinguishable in induction and maintenance to that of wildtype controls. **(M)**: Averaged potentiation levels of the last 5 min of LTP (55–60 min after TBS) were 147.31 ± 3.79% in wildtype slices compared to 141.59 ± 2.66% in APLP1-KOs (Student’s *t*-test, ^ns^p = 0.23). **(N)**: Neuronal excitability was comparable at all stimulus intensities (25–250 μA) between genotypes. **(O)**: Analyzing the Input–Output (IO) strength revealed no alterations between groups at any FV amplitude. **(P)**: PPF was unaltered between wildtype and APLP1-KO mice. Data information: *n* = number of neurons, N = number of animals. Age of animals **(B)–(K)**: 4–5 months. Age of animals **(L)–(P)**: 3–4 months. Data are represented as mean ± SEM.

Consistent with previous studies (Heber et al., 2000; Steubler et al., 2021), brain slices of APLP1-KO mice (age at analysis: 5–6 months) showed no gross morphological abnormalities in cortex and hippocampus (Figure 1A) and no astrogliosis and microgliosis as unspecific signs of neurodegeneration. As analysis of APP-KO mice had previously revealed impairments in neuronal morphology of cortical and hippocampal pyramidal cells (Seabrook et al., 1999; Lee et al., 2010; Tyan et al., 2012; Weyer et al., 2014), we first evaluated spine density as a correlate of excitatory synapse numbers. To this end CA1 neurons were filled with biocytin and stained with Alexa594-conjugated Streptavidin. Spine density analysis (age: 4–5 months; see Figure 1B for examples of dendritic segments) revealed no significant differences between APLP1-KO CA1 pyramidal cells, as compared to wild type (WT) neurons in both apical (1.24 ± 0.05 in APLP1-KO vs. 1.30 ± 0.05 in WT, ns, see Figure 1C) and basal dendrites (1.54 ± 0.05 in APLP1-KO vs. 1.58 ± 0.08 in WT, ns, see Figure 1D).

Subsequently, biocytin filled neurons were imaged and their dendritic tree was reconstructed for Sholl analysis (Figures 1E–K). Due to their different connectivity, the apical and basal dendrites of CA1 neurons were analyzed separately. Overall, pyramidal CA1 neurons of APLP1-KO mice showed no significant alteration in the total dendritic length of apical (3,246 ± 197 μm for APLP1-KO vs. 3,333 ± 179 μm for WT, see Figure 1F) or basal dendrites (1,399 ± 165 μm for APLP1-KO vs. 1,330 ± 125 μm for WT, see Figure 1G), similar maximal extension of apical dendrites (Figure 1H) and similar number of primary basal dendrites (Figure 1I).

Next, we performed a more detailed analysis by plotting the dendritic length (measured within circles centered on the soma) against the distance from the soma. In this analysis, an increased dendritic length per Sholl sphere corresponds to an increase in dendritic complexity. Apical dendrites of APLP1-KO CA1 neurons showed reduced branching towards more distant dendritic segments, with a significant decrease in complexity in dendritic segments at 480 μm from the soma (200.8 vs. 84.18 μm, Figure 1J). In contrast to this rather subtle difference in branching observed for apical dendrites, basal dendrites of CA1 neurons from APLP1-KO mice showed no difference in dendritic complexity (Figure 1K).

### APLP1-KO mice exhibit normal basal synaptic transmission and no alterations in synaptic plasticity

To study potential functional differences at the level of the hippocampal network we performed extracellular field recordings in acute hippocampal slices from mice of both genotypes. We first assessed long-term potentiation (LTP), a cellular process believed to underly learning and memory (Bliss and Lomo, 1973; Korte and Schmitz, 2016). After 20 min of stable baseline recording LTP was induced by Thetaburst stimulation (TBS) of the Schaffer collaterals and monitored for 60 min (Figure 1L). LTP was not significantly altered between APLP1-KO and WT controls resulting in closely overlapping LTP curves shortly after TBS and during the LTP maintenance phase (Figure 1L). Averaged potentiation levels of the last 5 min of LTP (t75-80, 55–60 min after TBS; see Figure 1M) were 147.31 ± 3.79% in wildtype control slices compared to 141.59 ± 2.66% in APLP1-KOs (Student’s t-test, *p* = 0.23). We also examined basal synaptic transmission and studied pre- and postsynaptic functionality. No significant differences between genotypes were detected when comparing the strength of fEPSP responses resulting from defined, increasing stimulus intensities (Figure 1N). Likewise, we failed to detect significant differences when correlating fiber volley amplitudes with fEPSP responses (input–output curves; Figure 1O), together indicating no major alterations at the postsynaptic side. Finally, to study putative presynaptic changes in APLP1-KO mice, we investigated short-term plasticity using the paired pulse facilitation (PPF) paradigm. APLP1-KO mice revealed no significant alteration in facilitation, when analyzing the ratio of fEPSP slopes resulting from two closely separated afferent stimuli (Figure 1P). Together, these data suggest normal basal synaptic transmission and synaptic plasticity in the hippocampus of mice lacking APLP1.

### APLP1-KO mice show deficits in grip strength and impairments in locomotor and exploratory activity

**Figure 2:**
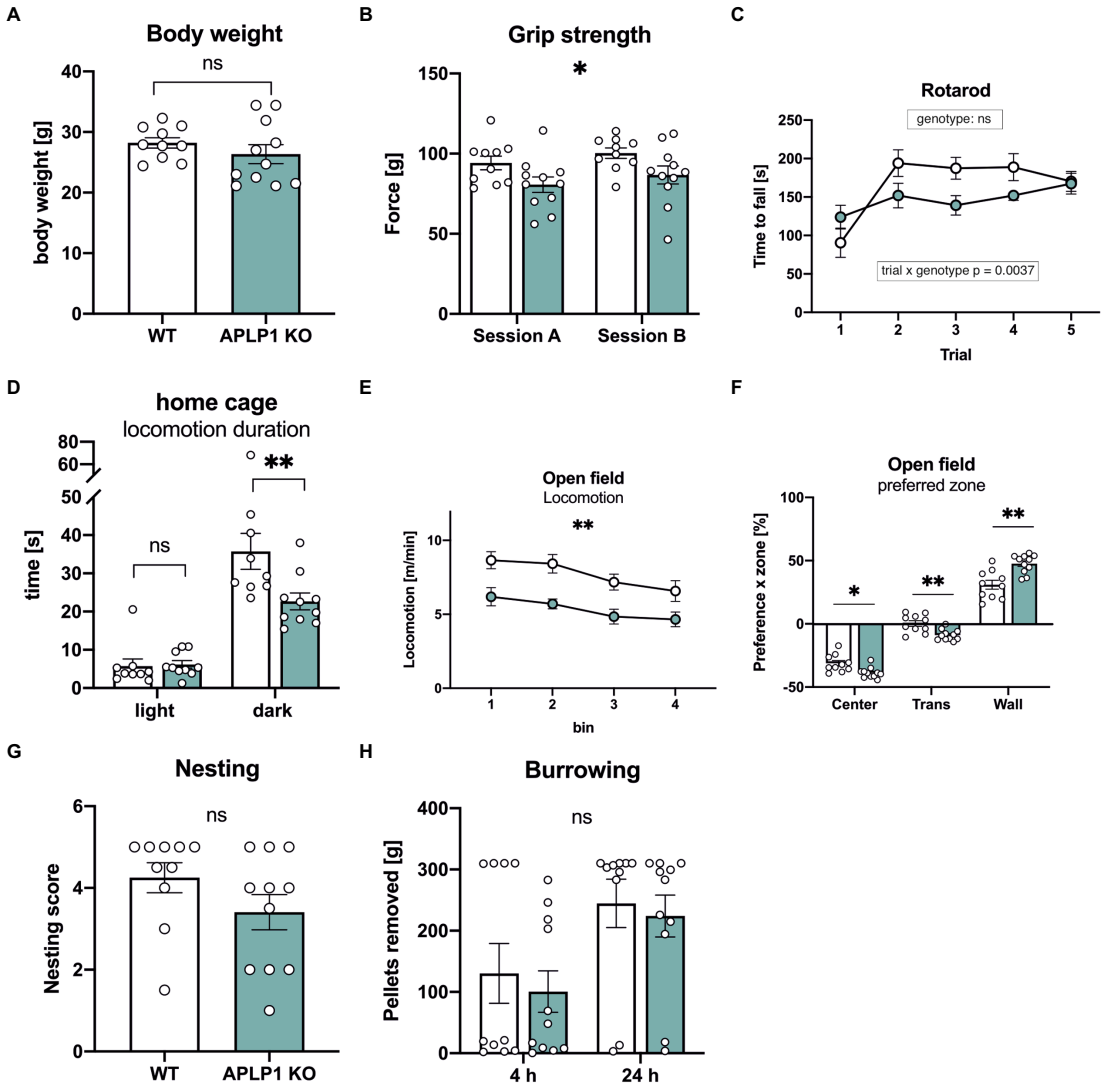
APLP1-KOs exhibit small impairments in innate and locomotor behavior. **(A)**: Bodyweight of APLP1-KOs is not different from wildtype controls (unpaired Student’s t-test, ^ns^p = 0.3272). **(B)**: Grip force of APLP1-KO animals is slightly reduced comparted to wildtype controls in two test sessions. [geno *F* (1, 19) = 5.079, *p* = 0.0362; session *F* (1, 19) = 5.282, *p* = 0.0331; session × geno *F* (1, 19) = 7.387e-005 ns]. **(C)**: Rotarod performance is comparable between APLP1-KO animals and wildtype controls. [geno *F* (1, 19) = 1.768 ns; trial *F* (2.875, 54.63) = 11.18, *p* < 0.0001; trial × geno *F* (4, 76) = 4.259, *p* = 0.0037]. **(D)**: Locomotion in the home cage. APLP1-KOs showed reduced activity in the dark phase. [geno *F* (1, 17) = 3.615, *p* = 0.0743; phase *F* (1, 17) = 144.8, *p* < 0.0001; phase × geno *F* (1, 17) = 12.29, *p* = 0.0027]. **(E)**: Open Field activity of APLP1-KOs is reduced compared to controls during exploration of a novel open field arena [geno *F* (1, 19) = 13.38 *p* = 0.0017; bin *F* (2.186, 41.54) = 11.20, *p* < 0.0001; bin × geno *F* (3, 57) = 0.4333, ns]. **(F)**: Open Field, APLP1-KOs showed an increased avoidance of the center field and a higher preference for the wall zone. [zone *F* (1.317, 25.03) = 387.5, *p* < 0.0001; zone × geno *F* (2, 38) = 14.60, *p* < 0.0001]. **(G)**: The nest score of APLP1-KOs is not significantly different from that of wildtype controls (unpaired Student’s t-test, ^ns^p = 0.1577). **(H)**: Burrowing: Pellets removed from tube after 4 and 24 h. Maximum to be removed = 310 g. Burrowing behavior was not significantly different between genotypes [geno *F* (1, 19) = 0.2828, ns; time *F* (1, 19) = 16.82, *p* = 0.0006; time × geno *F* (1, 19) = 0.02605, ns]. Data information: **(A–C; E–H)** number of animals: *N* = 10 WT, *N* = 11 APLP1-KO, age of animals 5 months. **(D)** number of animals: *N* = 9 WT, *N* = 10 APLP1-KO, age of animals 3–4 months. Data were analyzed using a mixed ANOVA model and are represented as mean ± SEM. ∗*p* < *p* < 0.05, ∗∗*p* < 0.01, ∗∗∗*p* < 0.001, ∗∗∗∗*p* < 0.0001, ns not significant.

We first studied body weight, that was unaltered in APLP1-KO mice (Figure 2A). As a baseline for subsequent cognitive tests, we started by examining the neuromotor performance of APLP1-KO mice. Compared to WT controls, APLP1-KO mice showed a small (about 14%) but significant deficit in forelimb grip strength (97.32 ± 3.05 vs. 83.66 ± 3.027, Figure 2B). On the rotarod that assesses motor coordination APLP1-KO mice showed slightly reduced motor learning during early trials (trial 2–4, see Figure 2C), but improved to levels undistinguishable from WTs at the final trial, with overall performance across trials not significantly different from WT animals (Figure 2C). Monitoring of the diurnal activity profile in a familiar home cage, we observed for APLP1-KO mice no difference in activity during the light phase, but severely reduced activity during the dark phase (35.75 vs. 22.66 s, Figure 2D). Similarly, also in the open field mutant mice showed deficits in basal locomotion throughout testing (7.709 vs. 5.349 m/min, Figure 2E). Both groups showed, however, habituation in the second 10 min of testing, with reduced activity as compared to the first 10 min. In addition, APLP1-KO mice spent more time along the wall of the open field arena (Figure 2F) at the expense of time spent in the center or intermediate zones, which may indicate reduced exploratory activity or increased anxiety. Next, mice were tested in the nesting and burrowing paradigms, two innate species-typic behaviors that are highly sensitive to hippocampal dysfunction (Deacon, 2006a, b). In both tests, APLP1-KO mice showed no significant impairments (Figures 2G, H).

### APLP1-KO mice show no significant deficits in spatial working memory

**Figure 3:**
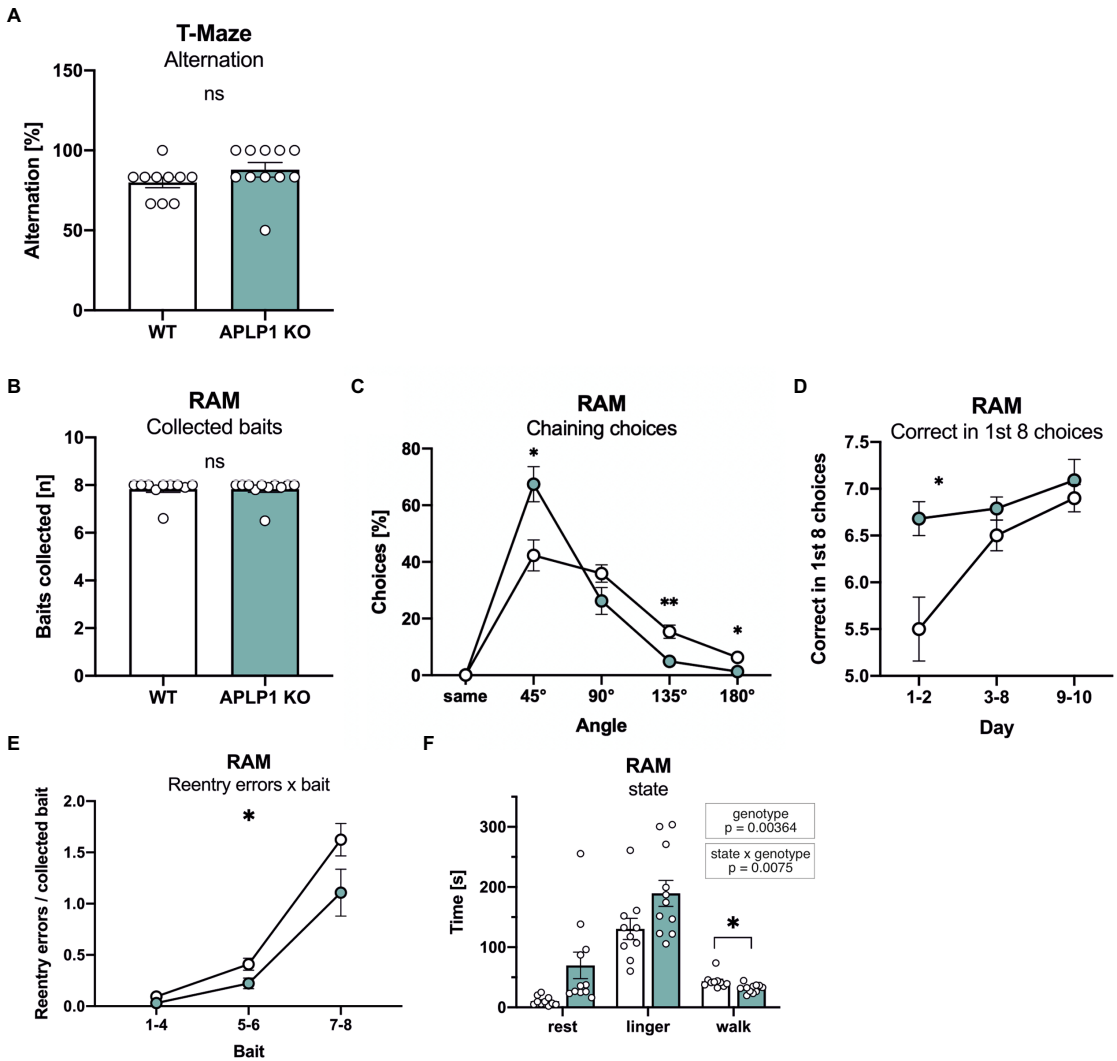
APLP1-KO mice exhibit normal short-term memory but use non-spatial search strategies more frequently. **(A)**: T-Maze alternation test shows no significant difference between genotypes (Mann–Whitney test, ^ns^p = 0.1149). **(B)**: Radial Arm Maze (RAM). Almost all animals from both genotypes collected all baits during the test (Mann–Whitney test, ^ns^p = 0.8565). **(C)**: RAM, APLP1-KOs showed a higher tendency for chaining choices than wildtype controls. [geno *F* (1, 19) = 1.791, ns; angle *F* (1.141, 21.68) = 74.31, *p* < 0.0001; angle × geno *F* (4, 76) = 7.631, *p* < 0.0001]. **(D)**: RAM, during training, APLP1-KO mice made more correct among the first 8 choices than wildtype controls, especially at the first two days of training. [geno *F* (1, 19) = 9.792, *p* = 0.0055; day *F* (1.405, 26.69) = 10.20, *p* = 0.0015; day × geno *F* (2, 38) = 3.660, *p* = 0.0352]. **(E)**: RAM, APLP1-KO animals made less reentry errors than controls during training. [geno *F* (1, 19) = 5.474, *p* = 0.0304; bait *F* (1.110, 21,09) = 74.62, *p* < 0.0001; bait × geno *F* (2, 38) = 2.131 ns]. **(F)**: RAM, APLP1-KO mice spent less time walking, but more time resting and lingering than wildtype controls [geno *F* (1, 19) = 5.069, *p* = 0.00364; state *F* (1.918, 36.43) = 65.03, *p* < 0.0001; state × geno *F* (2, 38) = 5.576, *p* = 0.0075 Data information: number of animals: *N* = 10 WT, *N* = 11 APLP1-KO, age of animals 5 months. Data were analyzed using a mixed ANOVA model and are represented as mean ± SEM. ∗*p* < 0.05, ∗∗*p* < 0.01, ∗∗∗*p* < 0.001, ∗∗∗∗*p* < 0.0001, ns not significant.

To assess possible deficits in learning and memory, mice underwent testing in a series of hippocampus dependent tasks. Working memory was studied in an unbaited T-maze that exploits the natural tendency of mice to show alternating visits between the two arms of the maze. Spontaneous alternation rates of APLP1-KO mice in the T-maze were indistinguishable from those of WT controls (Figure 3A).

Next, to assess spatial working memory, mice underwent testing in an 8-arm fully baited radial maze. Overall, APLP1-KO mice showed normal performance and collected on average all 8 baits, similar to WT mice (Figure 3B). More detailed analysis of performance over trials indicated that APLP1-KO mice have a stronger tendency to use a non-spatial strategy to solve the task. As such, they showed an increase in chaining choices visiting adjacent arms of the maze more frequently (Figure 3C), paradoxically earning them superior performance scores compared to WT mice during the first 2 days of training (Figure 3D). In both groups, the number of reentry errors increased with the number of baits already collected, reflecting the increasing challenge of working memory. APLP1-KO mice showed fewer reentry errors when collecting the last two baits (Figure 3E), likely due to their increased use of chaining choices. In this regard, and consistent with their impaired spontaneous locomotor activity, APLP1-KO mice also spend more time resting and lingering (walking: movement bouts >5 cm and > 8.5 cm/s, resting: >2 s <2.5 cm/s, lingering: rest + any deceleration deeper than 15 cm/s) in the center zone of the maze (Figure 3F), which may prevent them from performing inadvertent reentry errors (Figure 3F). In summary, APLP1-KO mice showed no impairments in spatial working memory, but showed a more pronounced use of a non-spatial strategy.

### APLP1-KO mice show no significant deficits in spatial reference memory

**Figure 4:**
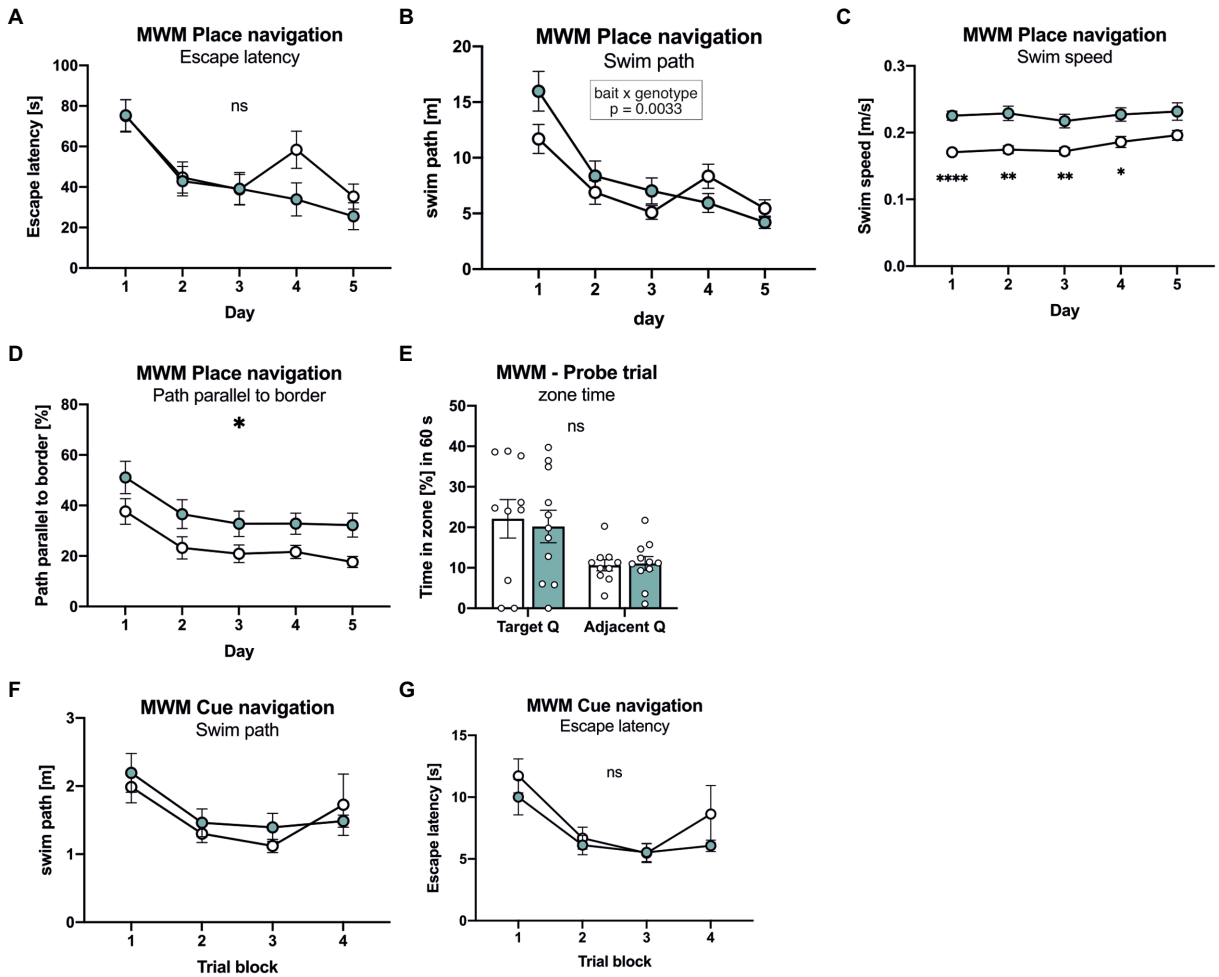
APLP1-KOs show no deficits in spatial learning and spatial reference memory. **(A)**: MWM, escape latency. During place navigation training, both genotypes showed evidence of learning. [geno *F* (1, 19) = 0.6471 ns; day *F* (3.455, 65.64) = 22.86, *p* < 0.0001; day × geno *F* (4, 76) = 2.228 ns]. **(B)**: Morris Water Maze (MWM), swim path. Both genotypes shortened their swim path over trials. Of note, wildtype controls showed a reversal effect while APLP1-KOs did not [geno *F* (1, 19) = 0.5215 ns; day *F* (2.871, 54.54) = 29.73, *p* < 0.0001; day × geno *F* (4, 76) = 4.321, *p* = 0.0033]. **(C)**: MWM, Swim speed of APLP1-KO animals was higher than in wildtype controls. [geno *F* (1, 19) = 20.47, *p* = 0.0002; day *F* (3.020, 57.39) = 3.562, *p* = 0.0194; day × geno *F* (4, 76) = 1.113 ns]. **(D)**: MWM, path parallel to the border. APLP1-KOs spent more time parallel to the border of the basin than wildtype controls. [geno *F* (1, 19) = 6.912, *p* = 0.0165; day *F* (2.286, 43.44) = 10.47, *p* = 0.0001; day × geno *F* (4, 76) = 0.07723 ns]. **(E)**: MWM, both genotypes preferred the trained target quadrant over the adjacent quadrant [geno *F* (1, 19) = 0.04617 ns; day *F* (1, 19) = 10.55, *p* = 0.0042; day × geno *F* (1, 19) = 0.1404 ns]. **(F)**: MWM, during cue navigation task, both genotypes do not differ in length of their swim path [geno *F* (1, 18) = 0.2342 ns; trial block *F* (2.596, 46.72) = 5.613, *p* = 0.0034; trial block × geno *F* (3, 54) = 0.5595 ns]. **(G)**: MWM, during cue navigation task, escape latency is not different between genotypes. [geno *F* (1, 18) = 1.139 ns; trial block *F* (2.581, 46.46) = 9.433, p = 0.0001; trial block × geno *F* (3, 54) = 0.5880 ns]. Data information: **(A)–(E)** number of animals: *N* = 10 WT, *N* = 11 APLP1-KO, age of animals 5 months. **(F, G)** number of animals: *N* = 10 WT, *N* = 11 APLP1-KO, age of animals 6 months. Data were analyzed using a mixed ANOVA model and are represented as mean ± SEM. ∗*p* < 0.05, ∗∗*p* < 0.01, ∗∗∗*p* < 0.001, ∗∗∗∗*p* < 0.0001, ns not significant.

Next, mice were tested in the Morris water maze place navigation task with 3 days of acquisition learning, followed by 2 days of reversal learning (Figure 4). Both groups of mice showed similar learning curves, with a similar decline of path length required to reach the platform (Figure 4B). Also escape latency was indistinguishable between groups during the acquisition phase (Figure 4A), despite slightly increased swim speed in APLP1-KO mice (Figure 4C). When the platform was relocated to the opposite quadrant on day 4 of testing APLP1-KO mice showed, unlike WT mice, no reversal effect (e.g., increase) in swim path (Figure 4A). To assess whether this may indicate less persistent searching in the trained quadrant or maybe due to differences in search strategy, we also analyzed the path length parallel to wall of the arena (Figure 4D). Indeed, APLP1-KO mice showed an increased percentage of their overall pathlength parallel to the border, which increases the likelihood to find the platform by chance. During the probe trial (first trial after platform relocation), both groups of mice showed a clear and statistically indistinguishable preference for the trained target quadrant (Figure 4E), indicating normal spatial reference memory in APLP1-KO mice. After place navigation, the platform was labelled with a flag to test for cued navigation. Both groups of mice showed similar performance with similar path length and escape latency, excluding visual problems in APLP1 mutant mice (Figures 4F, G).

**Figure 5:**
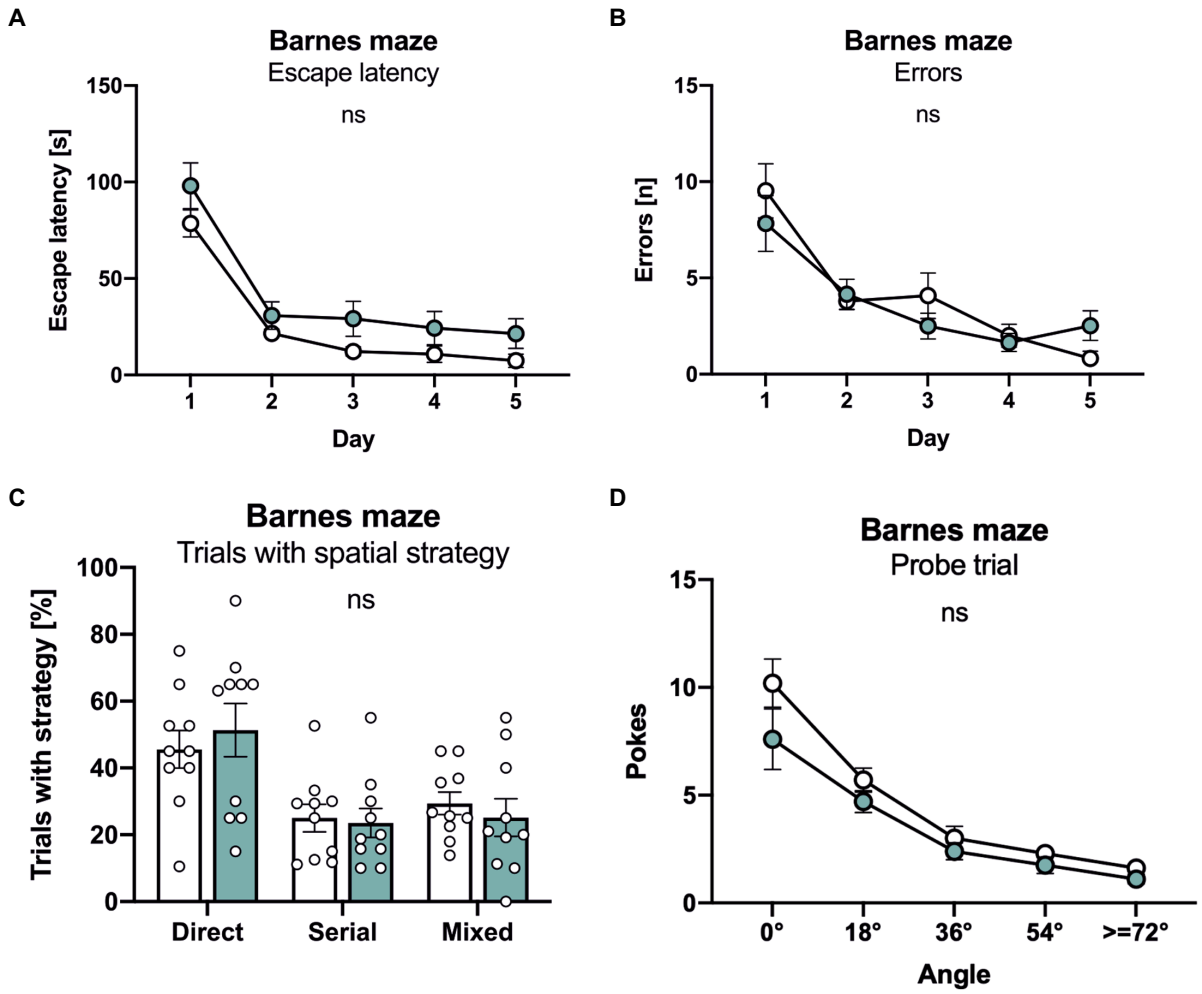
APLP1-KOs show no deficits in the Barnes maze. **(A)**: Barnes Maze (BM), throughout training, latency to escape the maze decreased similarly in APLP1-KO and control animals. [geno *F* (1, 18) = 4.271 ns; day *F* (2.347, 42.24) = 61.08, *p* < 0.0001; day × geno *F* (4, 72) = 0.2359 ns]. **(B)**: BM, APLP1-KO animals reduced their number of errors during training to the same extent as wildtype controls. [geno *F* (1, 18) = 0.1351 ns; day *F* (2.279, 41.03) = 29.47, *p* < 0.0001; day × geno F (4, 72) = 1.812 ns]. **(C)**: BM, trials of APLP1-KO mice with a direct spatial strategy was not significantly altered compared to wildtype controls. [strategy *F* (1.412, 25.42) = 7.964 ns; geno x strategy *F* (2, 36) = 0.3051 ns]. **(D)**: BM, during the probe trial, APLP1-KO animals performed as well as wildtype controls. [geno *F* (1, 18) = 3.113 ns; angle *F* (1.465, 26.37) = 54.39, *p* < 0.0001; angle × geno *F* (4, 72) = 1.129 ns]. Data information: number of animals: *N* = 10 WT, *N* = 11 APLP1-KO, age of animals 6 months. Data were analyzed using a mixed ANOVA model and are represented as mean ± SEM. ∗*p* < 0.05, ∗∗*p* < 0.01, ∗∗∗*p* < 0.001, ∗∗∗∗*p* < 0.0001, ns not significant.

Finally, mice underwent testing in the Barnes maze that consists of a brightly lit circular table with 20 circular holes around its circumference. Under one of the holes is an “escape box” which the mouse can reach through the corresponding hole on the table top. The test exploits the rodents’ aversion to open spaces, motivating them to seek shelter in the escape box. Both lines of mice rapidly learned the task and showed an indistinguishable decline in escape latency (Figure 5A). Further, no difference was detectable in the number of errors that declined in a similar manner during the five consecutive days of training (Figure 5B). As APLP1-KO had a higher tendency for a non-spatial strategy in the radial maze, we also analyzed the percentage of trials with a direct spatial strategy, versus serial and mixed approaches to find the escape hole. However, no significant difference between groups was detectable (Figure 5C). Likewise, during the probe trial, that was conducted 24 h after the last training session to test for spatial reference memory, APLP1-KO mice showed robust spatial retention of the goal position, with the number of correct pokes indistinguishable from WT mice (Figure 5D, correct pokes = angle 0°). We also failed to detect any difference in the number of pokes into adjacent or further distant holes between APLP1-KO and WT mice (Figure 5D). Taken together our data indicate largely normal cognition in APLP1-KO mice, with unimpaired spatial learning and normal short term and spatial reference memory.

## Discussion

### Brain architecture and neuronal morphology

In contrast to APP, that constitutes the precursor of Aβ peptides that accumulate as extracellular plaques in the brains of AD patients, the functions of APLP1 have been studied in much less detail. Recent studies however, revived the interest in APLP1, as it may serve as a (co)-receptor for α-synuclein fibrils (Zhang et al., 2021) and can be classified as a synaptic adhesion molecule (Schilling et al., 2017). Moreover, recently generated triple knockout mice lacking the whole APP gene family in the forebrain indicated an essential role of APP and the APLPs for brain development and function, as evidenced by severe impairments in synaptic function, plasticity and behavior, in particular completely disrupted learning and memory (Steubler et al., 2021). To generate these cTKO mice we had used a NexCre driver line that was crossed with APP^flox/flox^APLP2^flox/flox^APLP1-KO mice (designated as APLP1-KO) to generate conditional NexCre-cTKO mice and APLP1-KO littermate controls. Here, we now performed a detailed characterization of APLP1-KO mice as compared to WT mice to delineate potential APLP1 specific deficits.

We had previously shown that APLP1-KO mice show no gross alterations in brain morphology, with unaltered neocortical volume and normal layering of the cortex and hippocampus (Steubler et al., 2021). Unlike NexCre-cTKO mice, that showed a high incidence of callosal agenesis/dysgenesis, APLP1-KO mice were indistinguishable from WT controls (Steubler et al., 2021), indicating that APLP1 is not essential for normal development of this fiber tract connecting both brain hemispheres. Likewise, single deficiency of APP or APLP2 was not sufficient to cause agenesis of the corpus callosum, which points towards compensation within the gene APP family that may serve overlapping functions with regard to axonal outgrowth (Wang et al., 2017; Steubler et al., 2021).

Sholl analysis of hippocampal CA1 cells from APLP1-KO mice revealed rather subtle deficits in neuronal morphology, as evidenced by reduced dendritic branching in distal segments of the apical dendrite, while branching of basal dendrites and total dendritic length were unaffected. As APP family proteins have also been implicated in axonal outgrowth (Müller et al., 2017; Wang et al., 2017; Steubler et al., 2021) it is possible that the subtle reduction in dendritic complexity in distal regions of apical dendrites of APLP1-KO mice might also be related to changes in axonal projections from CA3, although this deserves further studies. Similar to APLP2-KO mice (Weyer et al., 2011; Midthune et al., 2012), young adult APLP1-KO mice did not show deficits in spine density at either apical or basal dendrites of CA1 pyramidal cells. Consitent with this, extracellular field recordings showed no alteration of basal excitatory synaptic transmission. Although APP and APLPs are upregulated postnatally during synaptogenesis and can potently induce pre-synaptic specializations *in vitro* (Schilling et al., 2017), our findings indicate that (1) they are not essential for initial synaptogenesis during development and/or (2) may have overlapping functions that require combined knockouts to reveal more severe phenotypes. Indeed, decreases in spine density were found in both aged APP-KO mice (Lee et al., 2010; Tyan et al., 2012), as well as in aged APLP1-KO mice together with reduced frequency of miniature EPSCs (Schilling et al., 2017). In contrast, APLP2 deficient mice had normal spine density even upon aging (Weyer et al., 2011; Midthune et al., 2012). Together, this indicates an essential role for spine maintenance for APLP1 and APP, although the underlying mechanism still needs to be unraveled. Together, normal brain architecture and only subtle alterations in neuronal morphology are also consistent with largely normal cognition in young APLP1-KO mice (see below). Consistent with overlapping physiological functions in the APP family, mice with brain-specific combined APP/APLP2 double KO mice revealed pronounced impaired spine density and morphology already in young adult mice *in vivo* (Hick et al., 2015; Richter et al., 2018; Steubler et al., 2021) that was associated with impaired LTP, learning and memory.

### Neuromotor functions and cognition

Our analysis of APLP1-KO mice indicated normal motor learning and coordination on the accelerating rotarod, but subtle deficits in grip strength, similar to APP-KO mice (Ring et al., 2007). Although newborn APLP1-KO mice exhibit, like APP-KOs, normal neuromuscular innervation and morphology with unaltered size of axonal presynaptic terminals and postsynaptic boutons (Klevanski et al., 2014), this does not exclude functional impairments. In this regard, electrophysiological analysis in neonatal (p18-22) APP-KO mice showed that grip strength deficits are associated with increased depression of synaptic transmission at the neuromuscular junction after high frequency stimulation of the phrenic nerve (Yang et al., 2007). This decrease in maximal grip strength of APLP1-KO mice contrasts, however, with an increase in swim speed during water maze testing, although the underlying reason for this discrepancy is currently unclear. Further, locomotor activity of APLP1-KO mice was impaired in the home cage and the open field, indicating together subtle impairments in neuromotor function in the absence of APLP1.

Interestingly, APLP1-KO mice proved unimpaired in all cognitive tasks (T maze, radial maze, Morris water maze, Barnes maze), as well as in nesting and burrowing tasks that are sensitive to hippocampal dysfunction. As such, they showed normal escape latency and normal probe trial performance both in the Barnes maze and in the Morris water maze, indicating normal spatial learning and spatial reference memory. This is further corroborated by normal synaptic plasticity. Similarly, performance of APLP1-KO mice on the radial- and T-mazes suggest normal spatial working memory. Refined analysis revealed increased use of a non-spatial chaining strategy by APLP1-KO mice both in the water-maze and radial-maze. While this could in principle reflect compensation of a subtle memory deficit, intact performance in the water-maze probe trial and in the T-maze - that does not allow chaining - render this unlikely and speak in favor of mere motivational differences. It will now be interesting to see whether aged APLP1-KO mice may develop behavioral impairments as observed for APP-KO mice (Dawson et al., 1999; Seabrook et al., 1999; Ring et al., 2007). In contrast to APP-KO mice, however, that not only showed aged dependent deficits in learning but also in synaptic plasticity at the CA3/CA1 pathway (Dawson et al., 1999; Seabrook et al., 1999; Ring et al., 2007), LTP impairments were absent in aged APLP1-KO mice (Schilling et al., 2017), indicating nonoverlapping specific functions for APP and APLP1 with regard to synaptic plasticity. This may be due to the absence of the CTα16 domain, located at the very C-terminus of APPsα, that was recently identified as the major LTP-enhancing region (Hick et al., 2015; Richter et al., 2018; Morrissey et al., 2019) and is not conserved in APLP1 and APLP2. In summary, our study indicates that APP family members serve both distinct and overlapping functions that are essential for nervous system development, synaptic plasticity and behavior. When targeting APP in the course of therapeutic intervention for AD, or APLP1 to inhibit α-synuclein propagation in Parkinson’s Disease, it will therefore be crucial to avoid compromising shared physiological functions within the APP family.

## Materials and methods

### Mice

APLP1-KO mice used in this study carry in addition floxed APP and APLP2 alleles and express APP and APLP2 at wild type level (Heber et al., 2000; Mallm et al., 2010). Genotyping was performed as described (Heber et al., 2000; Mallm et al., 2010). These APP^flox/flox^APLP2^flox/flox^APLP1-KO mice (referred to as APLP1-KO mice) had been generated as controls in a previous study of conditional APP/APLP1/APLP2 cTKO mice (Steubler et al., 2021). Here, we studied APLP1-KO mice as compared to C57Bl/6 J mice as the age-matched control group (further referred to as WT). Experiments involving animals were performed in accordance with the guidelines and regulations set forth by the German Animal Welfare Act, the Regierungspräsidium Karlsruhe and the Niedersächsiches Landesamt für Verbraucherschutz und Lebensmittelsicherheit Germany. Behavioral procedures were approved by the Veterinary Office of the Canton of Zurich (license ZH044/15, #26394). Animals were housed in the same room with a 12 h/12 h light/dark-cycle in Makrolon Type II (360 cm^2^) cages with standard bedding, either alone or in groups, and had *ad libitum* access to standard chow and water.

### Neuronal morphology and spine density analysis

CA1 pyramidal neurons used for morphological analysis were filled with a solution containing 0.1–0.5% biocytin (Sigma Aldrich) through a patch pipette. Acute slices were fixed in 4% Histofix (Carl Roth). After 2–10 days, the slices were washed in 1x PBS (phosphate-buffered saline) for 3× 10 min. Permeabilization was performed for 1 h in 0.2% PBST (0.2% Triton X-100 in 1x PBS). Slices were stained overnight with Alexa 594-conjugated Streptavidin directed against biocytin (Life Technologies). On the next day, the slices were washed again for 3× 10 min in 1x PBS. After air-drying the slices at RT for 1 h, they were mounted with a coverslip in ProLong Gold Antifade (Life Technologies).

#### Image acquisition

Images of filled neurons were acquired at the inverted fluorescence microscope Axio Observer Z1 using Plan Apo 20x/0.8 DICII and Plan Apo 63x/1.4 Oil DICII objectives (Zeiss). Overview images of the whole neuron for reconstruction were taken with a 20x objective and a z-step size of 0.5 μm. Basal and apical dendrites were imaged individually with two overlapping stacks. More detailed images of basal and apical dendritic segments for spine density analysis were acquired with a 63x oil objective and a z-step size of 130 nm. Exposure time was individually set for each cell so that the complete range of the grayscale was used.

#### Neuronal morphology and spine counts

Sholl analyisis and spine density analysis was performed as decribed (Steubler et al., 2021). Biocytin filled hippocampal CA1 neurons were manually reconstructed using the Neurolucida software (MicroBrightField) by an experimenter blind to genotype. Neurons were only included in Sholl analysis if they showed a completely filled apical or basal tree and well-defined dendritic endings. The morphometric Sholl analysis was done using the NeuroExplorer software (MicroBrightField). In short, a series of concentric spheres (centered on the soma) was drawn with an intersection interval of 30 μm and the number of dendrites crossing each sphere as well as the dendritic length in between each sphere was calculated. This analysis was done separately for basal and apical dendrites of CA1 pyramidal cells and was plotted against the distance from the soma.

For evaluation of basal dendritic spine density, at least 3 different dendritic segments of the basal dendritic arbor were imaged. They had to fulfill the following criteria: (1) Lie mostly horizontally to the slice surface, (2) be at least 20 μm away from the soma, (3) have a comparable thickness. The minimum basal dendritic length imaged per neuron was 100 μm. For evaluation of midapical dendritic spine density, at least 3 different dendritic segments of the apical tree were imaged. Midapical was defined as the middle third of the length of the apical dendrite measured from the origin of the apical dendrite from the soma to the endpoint of the tufts. Dendritic segments used for evaluation had to fulfil the following criteria: (1) be of second or third order to assure comparable shaft thickness, (2) lie in the middle third of the main apical dendrite (3) be longer than 10 μm. The minimum midapical dendritic length imaged per neuron was 100 μm. Files in the zvi format were imported into ImageJ (NIH) using the BioFormats Importer. After adjusting, images were saved in the TIFF format. Dendritic spines were manually counted using the Neurolucida and NeuroExplorer software (MicroBrightField) following the criteria of Holtmaat (Holtmaat et al., 2009) with minor modifications: (1) All spines that protruded laterally from the dendritic shaft and exceeded a length of 0.4 μm were counted. (2) Spines that protruded into the z-plane were only counted if they exceeded the dendritic shaft more than 0.4 μm to the lateral side. (3) Spines that bisected were counted as two spines. (4) Spines had to be at least 10 μm away from branching points and the soma. Spine density was expressed as spines per μm of dendrite. Prior to statistical analysis and blind to genotype, neurons were excluded if the image quality (poor signal to noise ratio) was not sufficient for counting of spines. Data acquisition and analysis were performed blind to genotype.

#### Preparation of acute hippocampal slices and extracellular field recordings

Extracellular recordings were performed on acute hippocampal slices of WT littermates and APLP1 KO animals (*N* = 5), as previously described (Schilling et al., 2017; Steubler et al., 2021). Acute hippocampal transversal slices were prepared from individuals at an age of 3–4 months Mice were anesthetized with isoflurane and decapitated. The brain was removed and quickly transferred into ice-cold carbogenated (95% O_2_, 5% CO_2_) artificial cerebrospinal fluid (ACSF) containing 125.0 mM NaCl, 2.0 mM KCl, 1.25 mM NaH_2_PO_4_, 2.0 mM MgCl_2_, 26.0 mM NaHCO_3_, 2.0 mM CaCl_2_, 25.0 mM glucose. The hippocampus was sectioned into 400 μm thick transversal slices with a vibratome (Leica, VT1200S) and maintained in carbogenated ACSF at room temperature for at least 1.5 h before transferred into a submerged recording chamber. Slices were placed in a submerged recording chamber and perfused with carbogenated ACSF (32°C) at a rate of 1.2 to 1.5 ml/min. Field excitatory postsynaptic potentials (fEPSPs) were recorded in stratum radiatum of CA1 region with a borosilicate glass micropipette (resistance 2–5 MΩ) filled with 3 M NaCl at a depth of ~150–200 μm. Monopolar tungsten electrodes were used for stimulating the Schaffer collaterals at a frequency of 0.1 Hz. Stimulation intensity was adjusted to ~40% of maximum fEPSP slope for 20 min baseline recording. LTP was induced by applying theta-burst stimulation (TBS: 10 trains of 4 pulses at 100 Hz in a 200 ms interval, repeated 3 times) and recorded for 60 min.

Basal synaptic transmission properties were analyzed *via* input–output-(IO) measurements and short-term plasticity was examined *via* paired pulse facilitation (PPF). The IO- measurements were performed either by application of defined current values (25–250 μA) or by adjusting the stimulus intensity to certain fiber volley (FV) amplitudes (0.1–0.8 mV). PPF was performed by applying a pair of two closely spaced stimuli in different inter-stimulus-intervals (ISI) ranging from 10 to 160 ms.

### Behavioral analysis

#### Neuromotor behavior and cognitive tests

##### Animals

Twenty-one Mice were tested in total: 11 APLP1 KO animals (APP ^flox/flox^/APLP2 ^flox/flox^/APLP1^−/−^; 5 females, 6 males) and 10 WT controls (C57Bl/6 J; 2 females, 8 males). Animals were housed under a 12/12 h light–dark cycle (lights on at 20:00) in groups of 2–5, unless individual housing was required by experimental protocols or to prevent fighting. Testing occurred during the dark phase under dim light (approximately 22 lux) if not stated otherwise, identity of genotype was blinded to the experimenter. Mice were transferred to the testing room 30 min before testing. Procedures were approved by the Veterinary Office of the Canton of Zurich (license ZH044/15, #26394).

Animals were aged 5 months at the beginning of behavioral testing. Test sequence was as follows: Open field, grip test, rotarod, water maze place navigation, burrowing, nesting, T-maze, radial maze, Barnes maze and water maze cue navigation. Tests including recovery periods in-between lasted 7 weeks.

##### Homecage, diurnal, and repetitive behavior

Home cage activity was recorded as described previously (Madani et al., 2003; Steubler et al., 2021) using a cage rack equipped with one passive IR sensor per mouse (ActiviScope, New Behavior Inc.,).^1^ The sensors detected any locomotion and remained silent only when the mice were sleeping or grooming. Recording started after a habituation period of at least 18 h, and circadian activity profiles were calculated by averaging data from 11 recording days.

##### Open field

Activity was tested as described previously (Madani et al., 2003; Steubler et al., 2021). In brief, animals were tested on two consecutive days for 10 min in the open field, a circular arena of 150 cm in diameter. Mice were tracked using Noldus EthoVision 11.5 software.^2^ For analysis of movement patterns, the arena was divided into a wall zone (18% of surface, 7 cm wide), a center zone (50%), and a transition zone in between.

#### Motor behavior, grip strength

Forepaw grip strength was measured as described previously (Ring et al., 2007; Steubler et al., 2021). using a newton meter (max. Force: 300 g). Animals had to hold on a metallic bar (4 cm long, 2.5 mm in diameter) attached to the horizontally positioned newton meter. Mice were held by the tail and allowed to grasp the bar with both forepaws. They were then gently pulled back until they released the bar. Mice were tested on two consecutive days for five trials each. For analysis, values of maximal pulling force were averaged.

#### Motor behavior, RotaRod

The RotaRod (Ugo Basile, model 47,600, Comerio, Italy) consisted of a rotating drum with a minimum speed of 2 round per minute (rpm) to maximal 40 rpm. Rotation speed was increased linearly until maximum speed was reached after 290 s. Animals were tested as previously described (Steubler et al., 2021) for 5 sessions on the same day, each session was terminated once the animal fell down the drum, or after 300 s the latest. Time at which animals for the first time clung to the drum (full circle ride), and time and acceleration at which the animals dropped off the drum was evaluated. For analysis, values were averaged.

#### Species-specific behavior, burrowing

The burrowing test was done as described previously (Deacon, 2006b; Steubler et al., 2021). In short, a grey plastic tube was filled with 310 g standard diet food pellets and placed at a slight angle into a Type III standard mouse cage equipped with normal bedding, a mouse shelter and water *ad libitum*. The lower end of the tube was closed, resting on the cage floor. The open end was supported 3.5 cm above the floor by two metal bolts. At the beginning of the dark period, mice were placed individually in the test cages, which were placed in their familiar animal room. At 4 h, and again at 24 h after experimental start the amount of non-displaced food (food still in the tube) was weighted. Consumed food by the animals (2 ± 0.5 g) was a very small proportion of the 310 g available and approximately equal across groups.

#### Species-specific behavior, nesting test

Nest building was studied as described (Deacon, 2006b; Steubler et al., 2021). At the beginning of the dark phase, mice were placed in individual testing cages (Type II) in their familiar animal room containing regular bedding and a Nestlet of 3 g compressed cotton (Ancare, Bellmore, USA). After 24 h, the nest-building activity of the mice was assessed on a rating scale of 1 to 5: 1 = Nestlet >90% intact, 2 = Nestlet 50–90% intact, 3 = Nestlet mostly shredded but no identifiable nest site, 4 = identifiable but flat nest, 5 = crater-shaped nest. Remaining intact parts of the Nestlet were weighted.

#### Water maze place navigation

Place navigation was assessed as described previously (Ring et al., 2007; Steubler et al., 2021). A white circular pool (150 cm diameter) contained milky water (24–26°C). Acquisition training consisted of 18 trials (6 per day, inter-trial interval 30–60 min) during which the submerged platform (14×14 cm) was left in the same position. Trials lasted a maximum of 120 s. To monitor reversal learning, the platform was moved to the opposite position for 2 additional days of testing (6 trials per day). Trials were video-tracked using a Noldus EthoVision. Raw data were transferred to the software Wintrack for analysis.^3^ Results were plotted in bins of 3 trials. Passive floating episodes were defined as immobility or decelerations with speed minimum <0.06 m/s and removed from the data before calculating swim speed. A slightly modified version of Whishaw’s error was calculated as path (%) outside a 18.5 cm wide corridor connecting release point and goal. Cumulative search error was determined by summing the distances to target measured at 1 s intervals and subtracting value that would be obtained for an ideal direct swim. Finally, wall-hugging was quantified by time (%) spent in a 10 cm wide wall zone. The first 30 s of the reversal trial served as probe trial to test for spatial retention.

#### Water maze cue navigation

On two consecutive days, mice were tested with the cued variant of the Morris water maze. For this, the location of the platform was marked with a black-and-white striped inverted pyramid (height 11 cm, base of pyramid 11x11cm) above the water. Animals were again tested in 6 trials per day, position of the flagged platform changed with each trial. Trials were video-tracked and analyzed as in place navigation.

#### T-maze

Spontaneous alternation on the T-maze was assessed as described (Deacon and Rawlins, 2006; Steubler et al., 2021). The T-maze was made of grey PVC. Each arm measured 30×10 cm. A removable central partition extended from the center of the back-goal wall of the T to 7 cm into the start arm. This prevented the mouse from seeing or smelling the non-chosen arm during the sample run, thus minimizing interfering stimuli. The entrance to each goal arm was fitted with a guillotine door. Each trial consisted of an information-gathering, sample run, followed immediately by a choice run. For the sample run a mouse was placed in the start arm, facing away from the choice point with the central partition in place. The mouse was allowed to choose a goal arm and was confined there for 30 s by lowering the guillotine door. Then the central partition was removed, the mouse replaced in the start arm, and the guillotine door was raised. Alternation was defined as entering the opposite arm to that entered on the sample trial (whole body, including tail). Three trials were run per day with an inter-trial interval of approximately 60 min. Each mouse received 6 trials in total and for data analysis the percentage of correct choices was calculated.

#### Radial maze

The working memory procedure on the 8-arm radial maze was carried out as described previously (Weyer et al., 2011; Steubler et al., 2021). Eight arms (7 × 38 cm) with clear Perspex tunnels (5 cm high) extended from an octagonal center platform (diameter 18.5 cm, distance platform center to end of arm 47 cm). The maze was placed 38 cm above the floor in a room rich in salient extramaze cues (same room as for open field testing). At the end of each arm, a metal cup (3 cm diameter) was lowered 1 cm to floor recess containing one millet-seed as bait (total ca 0.05 g), thus mice could not see the bait without completely entering the arm. Prior to the test, mice were gradually reduced to 85% of their free-feeding body weight for 2 days using a premeasured amount of chow, body weight was measured daily and 85% body weight was maintained throughout the test period. Water was available *ad libitum*. One day before test begin, mice were placed for 10 min into the baited radial maze for habituation. For testing, each mouse performed 1 session per day of maximally 10 min or until all eight seeds were collected. Test duration was 10 days. Mice were released in the center platform, performance of the animals was video-tracked, first visits to each arm and consumption of seeds were recorded manually. Using the video-tracking information, we calculated the number of correct choices among the first eight, as well as the number of reentry errors as a function of trial and of baits already collected. In addition, preferences for arm visits were analyzed. Error-free trials with one visit to each of the eight arms yielded preferred arm visits of 12.5%, corresponding to chance level without a preference for any arm. Reentries into already visited arms would yield values between 12.5–25%. Scores higher than 25% indicated excessive entry into one particular, preferred arm.

#### Barnes maze

The Barnes maze was made of a circular arena (1 m in diameter), placed 64 cm above ground. Twenty holes (5 cm in diameter) were evenly distributed at the margin of the platform. A black escape/goal box attached to the underside of a hole, equipped with a ramp inside, provided easy access to the dark escape. Tests were run in a brightly lit room for 5 days as previously described (Steubler et al., 2021). Each day, animals were trained in 4 trials, 3 min each, with a fixed position of the escape box. On the last day, one additional trial without escape box (probe trial) was run for 3 min. For each trial, animals were placed under a circular opaque start box in the platform center for 30 s. Trial started with removing the start box. If the animal successfully escaped into the goal box, the start box was placed over the whole with the goal box for another 30 s to prevent re-emergence of the mouse. Mice that did not succeed in finding the escape box within the given time were gently guided to the escape box during the first day of testing. Trials were video-tracked. Tracking data were used to calculate start delay (trial start until exit of start area), escape latency (exit of start area until disappearance of the animal), and number of errors (nosepokes into incorrect holes until first poke into the correct hole). In addition, trials were categorized according to search strategy: direct (max 1 error with absolute deviation angle <27°), serial (no center crosses and > 33% pokes to consecutive holes) or mixed (all remaining trials). During the probe trial, pokes were categorized according to deviation from the target hole.

## Data availability statement

The raw data supporting the conclusions of this article will be made available by the authors, without undue reservation.

## Author contributions

UM and DW designed and conceived the study. SE performed Sholl analysis, spine density analysis, and interpreted data. MB and JE performed biocytin fillings of neurons. IA and DW conducted, analyzed, and interpreted neuromotor and cognitive behavioral experiments. SL performed electrophysiological recordings, analyzed data, and interpreted data with MK. UM wrote the manuscript with help and input from all authors. All authors approved the final manuscript.

## Funding

We acknowledge funding by the Deutsche Forschungsgemeinschaft (MU 1457/15–1 and MU 1457/17–1 to UM and KO 1674/28–1 to MK). DW is a member of the Neuroscience Center Zurich (ZNZ) and of the Zurich Center for Integrative Human Physiology (ZIHP).

## Conflict of interest

The authors declare that the research was conducted in the absence of any commercial or financial relationships that could be construed as a potential conflict of interest.

## Publisher’s note

All claims expressed in this article are solely those of the authors and do not necessarily represent those of their affiliated organizations, or those of the publisher, the editors and the reviewers. Any product that may be evaluated in this article, or claim that may be made by its manufacturer, is not guaranteed or endorsed by the publisher.

## Footnotes

^1^ www.newbehavior.com

^2^ www.noldus.com
